# Supplementary material for: How and When to End the COVID-19 Lockdown: An Optimization Approach
Source: Front Public Health. 2020 Jun 10;8:262. doi: 10.3389/fpubh.2020.00262 (PMC7298102; doi:10.3389/fpubh.2020.00262)
Supplement: Supplementary file 1 [file Data_Sheet_1.pdf]

Appendix 1: Optimal Control of Covid-19 Exit Strategy: Collating Available Parameter Estimates

Our system of equations includes 7 rate parameters (Table 1 & Table 3) and 8 state variables (Table 3) for which we collated rate and initial value estimates from the available scientific literature. This is not an exhaustive compendium of all available estimates and should not be considered as such, rather this represents a collection of estimates from key papers designed to provide informative / sensible ranges to model. Note, for some parameters estimates we also collated estimates for related parameters (e.g. R0 estimates for β), but these were **not** used directly as estimates for our parameters of interest. We also collated estimates for other important / interesting parameters not considered in the current iteration of our system (Table 4). Finally for each estimate, we provide the reference from which the estimate was obtained and encourage readers to navigate to the associated paper rather than pulling estimates directly from this document.

Table 1: Parameters in the Exit Strategy SEIR Model

Symbol	Definition
β	Transmission rate
μ	Natural death rate
σ	Incubation rate
c	Transmission-reduction (due to quarantine)
α	Disease-induced death rate
	Recovery rate
I_{Thresh}	Threshold number of infections

Table 2: Parameter estimates for the rate parameters in the Exit Strategy model from the Covid-19 literature.

Symbol	Definition	Estimate(s)	Source(s)
β	Transmission rate	(4)<br>Estimated time-varying R0 (R_t) from 4 datasets. R0 / R_t declined from 2.35 (95% CI 1.15 - 4.77) on January 16th (week prior to restrictions) to 1.05 (0.41 - 2.39) on January 31st 8 days after restrictions were imposed.<br><br>(9)<br>Estimated the transmission rate as 1/σ, where σ is the infectious period in days. This they pulled from a Gaussian distribution with mean = 4.5, SD = 1.<br><br>(10)<br>**Seasonality and Social Distancing**<br>Kissler et al. simulated a wintertime R0 between 2.2 and 2.6 and allowed R0 to vary between 60% (some seasonality) and 100% (no seasonality) during the summer. Social distancing was simulated by reducing R0 by a fixed proportion between 0% (no transmission) and 60. B is calculated as β(t) = R0(t), where = rate at which infectious individuals recover.	(4), (9), (10)

μ	Natural death rate	Saw something on twitter from the ONS imposing the covid-deaths onto the average weekly death rates for the UK - could try and dig out the weekly data in the absence of COVID-19 for this?<br><br>(10)<br>**Individual Death Rate**<br>Individuals died at rate μ such that the average lifespan was 1/μ = 80 years (could re-scale this for the UK perhaps?). Note that they fully susceptible individuals were born with the same rate so that the population size remained constant.	(10)
σ	Incubation rate	(2)<br>**Median Incubation Period**<br>Using 181 confirmed cases (25 countries, inc. China, and 25 mainland Chinese provinces) they calculated estimates for the incubation time of COVID-19:<br>*Full Dataset*<br>Median incubation period = 5.1 days (CI, 4.5 - 5.8)<br>*Exc. Chinese Cases*<br>Median incubation period = 5.7 days (CI, 4.9 - 6.8)<br>*Excluding non-Chinese cases*<br>Median incubation period = 5.5 days (CI, 4.4 - 7.0)<br><br>(3)<br>**Daily probability of exposed becoming infected**<br>Use an estimate for duration of incubation period (6.4 days from Backer et al.(more up to date estimates now exist) to calculate the daily probability of exposed individual becoming infectious (K):<br>K = 1 - exp(-1/dI), where dI = average incubation period = 6.4. Note that in their model, they calculate the number of exposed becoming infectious as follows … -(1 - K)E, which I'm unsure about - why not just … -KE?. I think it must be a typo in their Et+1 eq.<br><br>(7)<br>**Mean days from exposure to illness onset**<br>Uncorrected without Wuhan Residents:<br>Mean incubation period = 5 days (95% CI: 4.2 - 6)<br>Uncorrected with Wuhan Residents:<br>Mean incubation period = 5.6 days (95% CI: 5 - 6.3)<br>Corrected without Wuhan Residents:<br>Mean incubation period = 5.6 days (95% CI: 4.4 -7.4)<br><br>(8)<br>Mean incubation period = 6.4 days (95% CI: 5.6 - 7.7)<br>Range: 2.1 - 11.1 days (2.55h - 97.5th percentile)	(2), (3), (7)
c	Transmission-reduction	**Mean Rt Reductions**<br>Average posterior mean Rt across 11 posterior means of 1.43, a reduction of 64% compared to the pre-intervention values. (i.e. c = 0.64)	(11)
α	Disease-induced death rate	(1)<br>**Case Fatality Ratio**<br>Using data from China, calculated an overall case fatality	(1), (5), (11)

		rate = 1.38% (1.23% - 1.53%)<br>When stratified by age:<br>< 60 fatality rate = 0.32% (0.27 - 0.38 95%CI)<br>>60 fatality rate = 6.4% (5.7 - 7.2, 95%CI)<br>> 80 fatality rate = 13.4% (11.2 - 15.9, 95%CI)<br>**Infection Fatality Ratio**<br>Calculated an overall infection fatality ratio of 0.657% (0.389-1.33)<br>< 60 fatality rate = 0.145% (0.0883 - 0.317 95%CI)<br>>60 fatality rate = 3.28% (1.82 - 6.18, 95%CI)<br><br>(5)<br>**Case fatality rate**<br>Calculate mortality rate by dividing deaths at time t by number of confirmed infections on t-14 (maximum incubation period - bit dubious?).<br>Global case fatality rate converges on 5.7% (95% CI. 5.5 - 5.9)<br><br>(11)<br>**Mortality Rates**<br>Hubei:<br>Overall = 3.0% (2.6 - 3.4) (all people infected)<br>>80 years = 32.% (25.3 - 40.1)<br>Overall = 3.6% (3.1 - 4.1)(infected people with symptoms)<br>50-59 = 2,5% (1.9 - 3.1)<br>60-69 = 8.0% (6.6 - 9.5)<br>70-79 = 19.2% (15.8 - 22.9)<br>>80 = 39% (31.1 - 48.9)<br><br>Northern Italy:<br>Overall: 3.3% (2.0 - 4.7) (all people infected)<br>Overall = 4.0% (2.4 - 5.7)(infected people with symptoms)<br>50-59 = 1.0% (0.6 - 1.4)<br>60-69 = 5.4% (3.4 - 6.9)<br>70-79 = 35.7% (22/4 - 42.7)<br>>80 = 89% (56.2 - 99.6)	
[illegible]	Recovery rate	(3)<br>**Daily probability that an infected individual recovers**<br>Daily probability than an infected individual recovers = g = 1 - exp(-1/di), where di = average duration of infection = 3 or 7 days (estimates from Weolfel et al.).	(3)
I_{Thresh}	Threshold Number of Infected Individual	(6)<br>**Total Number of ICU Beds in the UK**<br>Davies et al. tallied the total number of ICU beds in Wales, England, Scotland and Northern Ireland (references 14-17 in their manuscript) to get an idea of the ICU capacity before efforts to enhance capacity began.. Total number of ICU beds in the UK = 4562.<br><br>(13)<br>**Surge Capacity of ICU Beds per 100,000 people, UK**<br>Only skimmed this, but Ferguson et al. appear to assume surge capacity of ICU beds in the UK to be 14 per 100,000 people. Given a UK population of 66.6 million, this gives (66,000,000 / 100,000) * 14 = 9240 beds(approx. twice the pre-covid ICU capacity estimated by Davies et al.)	(6), (13)

Table 3: Estimates for initial values for state variables

Definition	Estimate(s)	Source(s)
S_o		
E_o		
I_o	(12)<br>**Mean estimated proportion of the United Kingdom as of 28th March**<br>2.7% [95% CrI 1.2% - 5.4%]<br><br>**UK Government Coronavirus Cases and Deaths Tracker**<br>https://coronavirus.data.gov.uk/<br>As of 19th April 2020:<br>Total number of lab-confirmed cases: 120,067<br>Total number of COVID-19 associated deaths: 16,060<br>Latest daily number of lab-confirmed cases: 5850<br>Latest daily number of COVID-19 associated UK deaths: 596	(12)
R_o		
$S_{Q,0}$		
$E_{Q,0}$		
$I_{Q,0}$		
$R_{Q,0}$		
N_tot	**Office for National Statistics**<br>2018: 66, 435, 600	

Table 4: Estimates for other potentially interesting parameters not currently captured in our model

Definition	Estimate(s)	Source(s)
Mean time from disease onset to death	(1)<br>17.8 days (95% Credible Interval; 16.9 - 19.2)<br><br>(6)<br>Based on estimates from Linton et al. (2020) and Cao et al. (2020), Davies et al. pulled delay from onset to death (days) values from a gamma distribution with mu = 22, k = 22).<br><br>(7)<br>Uncorrected<br>15 days (95% CI: 12.8 - 17.5)<br>Corrected<br>20.2 days (95% CI: 15.1 - 29.5)	(1), (6), (7)
Mean time from disease onset to recovery	(1)<br>24.7 days (95% Credible Interval; 22.9 - 28.1)	(1), (7)
R0	(6)<br>They took estimates (median + 95% CIs) from 11 studies (see supplementary materials), matched a PERT distribution, and then sampled each to produce distriubtion of R0s with a mean of 2.68 +/	(6), (9), (12)

	0.57 (sd).<br><br>(9)<br>Used estimates of R0 from 2.25 to 2.75 based on available estimates<br><br>(12)<br>**Cross-country (11 EU Countries) estimated R0**<br>3.87 [3.01 - 4.66] (from about 17th February I think - not 100%	
Percentage of cases resulting in hospitalisation	(13)<br>Table 1 of Ferguson et al. gives age-stratified % symptomatic cases requiring hospitalisation and percent hospitalised cases requiring critical care. They also present an in-text estimate of 4.4% of infections total hospitalised.	(13)
Percentage of hospitalised cases requiring ICU	(13) + (14)<br>Both Davies et al. and Ferguson et al. assume that of all individuals hospitalised, 30% will require critical care. This appears to be based on the findings of (14), albeit this paper suggests 31% of 199 patients required non-invasive ventilation and 0.5% required invasive mechanical vemtilation..<br><br>(13)<br>Ferguson et al. adjust data from Verity et al. and give the following age-stratified % hospitalised cases requiring critical care<br>0-9: 5%<br>10-19: 5%<br>20-29: 5%<br>30-39: 5%<br>40-49: 6.3%<br>50-59: 12.2%<br>60-69: 27.4%<br>70-79: 43.2%<br>80+: 70.9%	(6), (13), (14)
Proportion of UK population classed as key workers	From the Institute for Fiscal Studies website:<br>https://www.ifs.org.uk/publications/14763<br><br>Using Labour Force Survey data from 2018/19, they estimate that 22% of working age individuals, equating to 7.1 million adults across the UK, are in the set of key-worker guidelines set out by the UK government.	

References

1. Verity, R., Okell, L.C., Dorigatti, I., Winskill, P., Whittaker, C., Imai, N., Cuomo-Dannenburg, G., Thompson, H., Walker, P.G., Fu, H. and Dighe, A., 2020. Estimates of the severity of coronavirus disease 2019: a model-based analysis. The Lancet Infectious Diseases.
2. Lauer, S.A., Grantz, K.H., Bi, Q., Jones, F.K., Zheng, Q., Meredith, H.R., Azman, A.S., Reich, N.G. and Lessler, J., 2020. The incubation period of coronavirus disease 2019 (COVID-19) from publicly reported confirmed cases: estimation and application. Annals of internal medicine.
3. Prem, K., Liu, Y., Russell, T.W., Kucharski, A.J., Eggo, R.M., Davies, N., Flasche, S., Clifford, S., Pearson, C.A., Munday, J.D. and Abbott, S., 2020. The effect of control strategies to reduce social mixing on outcomes of the COVID-19 epidemic in Wuhan, China: a modelling study. The Lancet Public Health.
4. Kucharski, A.J., Russell, T.W., Diamond, C., Liu, Y., Edmunds, J., Funk, S., Eggo, R.M., Sun, F., Jit, M., Munday, J.D. and Davies, N., 2020. Early dynamics of transmission and control of COVID-19: a mathematical modelling study. The lancet infectious diseases.
5. Baud, D., Qi, X., Nielsen-Saines, K., Musso, D., Pomar, L. and Favre, G., 2020. Real estimates of mortality following COVID-19 infection. The Lancet infectious diseases.

6. Davies, N.G., Kucharski, A.J., Eggo, R.M., Gimma, A., Edmunds, W.J. and CMMID COVID-19 Working Group, 2020. The effect of non-pharmaceutical interventions on COVID-19 cases, deaths and demand for hospital services in the UK: a modelling study. medRxiv.
7. Linton, N.M., Kobayashi, T., Yang, Y., Hayashi, K., Akhmetzhanov, A.R., Jung, S.M., Yuan, B., Kinoshita, R. and Nishiura, H., 2020. Incubation period and other epidemiological characteristics of 2019 novel coronavirus infections with right truncation: a statistical analysis of publicly available case data. Journal of clinical medicine, 9(2), p.538.
8. Backer, J.A., Klinkenberg, D. and Wallinga, J., 2020. Incubation period of 2019 novel coronavirus (2019-nCoV) infections among travellers from Wuhan, China, 20–28 January 2020. *Eurosurveillance*, *25*(5), p.2000062.
9. Lourenço, J., Paton, R., Ghafari, M., Kraemer, M., Thompson, C., Simmonds, P., Klenerman, P. and Gupta, S., 2020. Fundamental principles of epidemic spread highlight the immediate need for large-scale serological surveys to assess the stage of the SARS-CoV-2 epidemic. medRxiv.
10. Kissler, S., Tedijanto, C., Goldstein, E., Grad, Y. and Lipsitch, M., 2020. Projecting the transmission dynamics of SARS-CoV-2 through the post-pandemic period.
11. Hauser, A., Counotte, M.J., Margossian, C.C., Konstantinoudis, G., Low, N., Althaus, C.L. and Riou, J., 2020. Estimation of SARS-CoV-2 mortality during the early stages of an epidemic: a modelling study in Hubei, China and northern Italy. medRxiv.
12. Flaxman, S., Mishra, S., Gandy, A., Unwin, H., Coupland, H., Mellan, T., Zhu, H., Berah, T., Eaton, J., Perez Guzman, P. and Schmit, N., 2020. Report 13: Estimating the number of infections and the impact of non-pharmaceutical interventions on COVID-19 in 11 European countries.
13. Ferguson, N., Laydon, D., Nedjati Gilani, G., Imai, N., Ainslie, K., Baguelin, M., Bhatia, S., Boonyasiri, A., Cucunuba Perez, Z.U.L.M.A., Cuomo-Dannenburg, G. and Dighe, A., 2020. Report 9: Impact of non-pharmaceutical interventions (NPIs) to reduce COVID19 mortality and healthcare demand.
14. Cao, B., Wang, Y. and Wen, D., A trial of lopinavir-ritonavir in adults hospitalized with severe Covid-19 [published online ahead of print March 18, 2020]. N Engl J Med, https://doi. org/10.1056/NEJMoa2001282.
15. Jit, M., Jombart, T., Nightingale, E., Endo, A., Abbott, S. and Edmunds, W.J., 2020. Estimating number of cases and spread of Coronavirus disease 2019 (COVID-19) in the United Kingdom using critical care admissions, February to March 2020. medRxiv.
